# Emergence and expansion of carbapenem resistant enterobacterales in the Western Cape Province, South Africa

**DOI:** 10.1371/journal.pone.0309315

**Published:** 2024-08-26

**Authors:** Kedišaletše Moloto, Mae Newton-Foot, Andrew Whitelaw, Angela Dramowski

**Affiliations:** 1 Division of Medical Microbiology, Department of Pathology, Faculty of Medicine and Health Sciences, Stellenbosch University, Cape Town, South Africa; 2 National Health Laboratory Service, Tygerberg Hospital, Cape Town, South Africa; 3 Department of Paediatrics and Child Health, Faculty of Medicine and Health Sciences, Stellenbosch University, Cape Town, South Africa; Hawassa University College of Medicine and Health Sciences, ETHIOPIA

## Abstract

**Background:**

Carbapenem resistant Enterobacterales (CRE) have become established as leading pathogens in South African healthcare facilities. The aim of this study is to describe the epidemiology of CRE carriage and clinical infection episodes at healthcare facilities in the Western Cape Province of South Africa (2016–2020), and identify factors associated with mortality in CRE infected patients.

**Methodology:**

We used routine data from the Provincial Health Data Centre to track the emergence of CRE in healthcare facilities in the Western Cape Province of South Africa. We included all CRE episodes (clinical and carriage) at Western Cape hospitals (including day and inpatients) from 2016 to 2020 to determine the distribution of CRE, patient demographics and antibiotic resistance phenotypes. Logistic regression was performed to identify factors associated with mortality from clinical CRE episodes.

**Results:**

2242 CRE episodes (1580 [70.5%] clinical and 662 [29.5%] carriage) were identified. From these, 2281 CRE isolates were identified, with *Klebsiella* species predominating (1644, 72.1%). Affected patients had a median age of 31 (IQR 0–52) years, and 1167 (52.0%) were male. Most CRE episodes were recorded in central hospitals (70.0%, *p* < 0.001). Where outcome data was available, crude in-hospital mortality rates were 26.9% (371/1379) for CRE clinical episodes versus 6.4% (41/640) for CRE carriage episodes (*p* < 0.001). Factors that showed a statistically significant association with in-hospital mortality were female sex [adjusted odd ratio (aOR) 1.40 (95% confidence interval (CI) 1.09–1.560)], adult patients [aOR 1.76 (95% CI 1.20–2.57)], CRE isolation from a sterile specimen [aOR 0.41 (95% CI 0.32–0.53)], and >3 days between hospital admission and specimen collection [aOR 1.56 (95% CI 1.11–2.18)]

**Conclusions:**

CRE episodes at Western Cape healthcare facilities are concentrated at tertiary hospitals, with high case fatality rates in patients with clinical CRE episodes. Infection control interventions must be strengthened to reduce transmission of CRE, and to reduce infection risks.

## Introduction

Carbapenem-resistant Enterobacterales (CRE) are a major public health threat, ranking among the leading antimicrobial resistant (AMR) pathogens on the World Health Organization’s priority list [1]. CRE cause a range of infections including bacteraemia, pneumonia, urinary tract infections, and device-associated infections, all with substantial associated morbidity and mortality. However, CRE may also result in asymptomatic carriage [2].

Rising rates of CRE infection threaten the continued efficacy of currently available antibiotics [2]. The excess mortality rates associated with CRE may be due to delayed initiation of effective antibiotic therapy and the pharmacologic limitations of available treatment options [3], but may also reflect that sicker patients and those who have been hospitalised for longer are at higher risk of CRE infection. CRE are typically healthcare-associated pathogens, with risk factors for acquisition including extended hospitalisation and intensive care unit (ICU) stay, presence of indwelling devices, previous carriage or infection with CRE, and prior exposure to antibiotics [4].

Antimicrobial resistance (AMR) is a threat to public health and modern medicine. Infections due to resistant pathogens result in a significant global disease burden. In 2019, an estimated 1.27 million deaths were attributable to antibiotic-resistant bacteria. The highest AMR-attributable death rates observed in Sub-Saharan Africa were in 2019 at 27.3 deaths per 100 000 [5]. In South Africa, there were an estimated 9,500 deaths attributable to AMR and 39,000 deaths associated with AMR in 2019 [6]. The country experienced an alarming increase in CRE prevalence among *Klebsiella* spp., *Enterobacter* spp., *Escherichia*. *coli* and *Citrobacter* spp. isolates between 2012 and 2015 [7, 8]. CRE outbreaks are frequently reported, with progression to endemicity in many healthcare facilities [9, 10]. However, much of the CRE data from South Africa is limited to specific specimen types (usually blood cultures), to tertiary facilities, and/or to focal outbreaks [11–13]

Given the increasing burden of CRE in healthcare facilities nationally, similar increases in CRE infections and colonisation rates may be expected in the community, and in stepdown care or rehabilitation facilities [14]. There is little local data describing this aspect of CRE epidemiology. Surveillance is important to track CRE burden in communities and healthcare facilities, at local institutional, provincial and national level. In this study we utilize routine clinical and laboratory datasets to describe the epidemiology of CRE carriage and clinical infection episodes at healthcare facilities in the Western Cape Province of South Africa (2016–2020), and identify factors associated with mortality in CRE infected patients.

## Methodology

### Study design and study site

A retrospective descriptive study was conducted to describe the epidemiology of both clinical and carriage CRE episodes from all healthcare facilities in the Western Cape Province of South Africa (https://www.westerncape.gov.za/static/health-facilities/) In 2020, the Western Cape province had approximately seven million inhabitants with three quarters of them reliant on public healthcare services [15]. Routine laboratory data from the National Health Laboratory Service (NHLS) was analysed from inpatient and outpatient public healthcare facilities including central, regional and district hospitals, specialised hospitals, and community-based clinics [16].

### Data collection

Clinical and laboratory data was obtained from the Provincial Health Data Centre’s (PHDC) Single Patient Viewer (SPV). SPV is a web-based electronic health portal that integrates clinical data for individual patients both longitudinally and cross-sectionally, in tabular and graphical views, to assist clinicians with rapid information discovery [15]. Data was extracted by a PHDC analyst for all patients with a positive CRE culture seen at any public healthcare facility in the Western Cape between 2016 and 2020.

Clinical and carriage episodes were defined based on the test-type, either “culture” for clinical or “CRE-screen” for carriage. Carriage isolates were from samples (predominantly rectal swabs) sent to the laboratory as part of active screening for CRE carriage, while clinical isolates were from any specimen submitted as part of the routine clinical assessment for infection in the patient. Sterile sites were defined as those in which microbes do not exist as commensals when in a healthy state, including tissue, aspirate, ascitic fluid, pleural fluid, pericardial fluid, bile, blood and cerebrospinal fluid. Non-sterile sites often contain commensal or transient microbes and included urine, bronchoalveolar lavage, central venous catheters, superficial wound swabs, stool, rectal swab, sputum, tracheal aspirate [17]

Clinical variables retrieved were sample collection date, age at sample collection, sex, date of admission or first visit to hospital, date of discharge (unless patients were day cases and not admitted), facility name, ward (medical or surgical), diagnosis, date of diagnosis, clinical outcome (death or discharge), and date of clinical outcome. Three age categories were used: neonate (0–28 days), child (>28 days to 13 years); adult (>13 years). These are the ages used by the provincial health services to determine whether a patient is admitted to a neonatal, paediatric or adult ward. Laboratory variables extracted were test type, specimen type, organism isolated and antibiotic susceptibility testing (AST) profile.

### Definition of CRE episodes

Members of the Enterobacterales order that were included in the analysis as CRE were *Citrobacter* spp., *Enterobacter* spp., *E*. *coli*, *Klebsiella* spp., *Serratia marcescens*, *Morganella* spp. and *Providencia* spp. that were non-susceptible to ertapenem.

The primary clinical episode was defined as the first CRE positive clinical culture per unique patient. Patients with clinical CRE episodes were re-included in the analysis if a different CRE species was identified at any point after the primary event, or if the same CRE species was re-isolated from a clinical specimen after >90 days [18]. Patients with a CRE carriage episode were re-included if there was an additional carriage episode >12 months from the first episode. If a patient had a clinical and a carriage episode, both were included.

If there were two different CRE species from different samples collected on the same day or at any point following the primary episode, these were considered as two different episodes, whereas, if they were from the same sample, they were considered as one episode. The different species were counted separately from the episodes.

### Data analysis

Descriptive analyses (simple proportions, mean [SD] for normally distributed data, median [IQR] for non-parametric data) were performed to describe the distribution of CREs, patient demographics, infection types, pathogen and antibiotic resistance profiles, and associated laboratory markers of infection.

In addition, we reported in-hospital outcome and proportion of deaths likely to be directly attributable to CRE infection, which was defined as death within 72 hours of clinical culture submission [19]. Student t-tests were used to compare continuous data and the Pearson chi-square test to compare categorical data. Logistic regression analyses were performed to determine factors associated with mortality from CRE infection. A *p*-value <0.05 was considered statistically significant. Statistical analyses were done using the Stata statistical software package v18.0 (StataCorp, USA).

### Ethical considerations

This study was approved by the Stellenbosch University’s Health Research Ethics Committee (HREC) (ethics reference: S21/01/005) as well as the Provincial Health Data Centre (study #1507). Data from the analyst was received on the 18^th^ May 2022. The data was non-identified individualised data, and the authors did not have access to information that could identify individual participants during or after data collection. All patient data was de-identified from the outset and only the investigators had access to the databases.

## Results

### CRE episodes

A total of 10 592 CRE were isolated from laboratory specimens submitted to the NHLS from Western Cape healthcare facilities from 2016 to 2020. Following deduplication, 2242 CRE episodes were identified, including 1580 (70.5%) clinical episodes from 1457 patients, and 662 (29.5%) carriage episodes from 639 patients (Table 1). In total, 2096 patients were included with a clinical and/or a carriage episode. The largest number of clinical and carriage episodes occurred in 2019 (n = 930, 41.5%) and 2020 (n = 766, 34.2%). Prior to 2019, CRE cases were sporadic with very low rates of laboratory screening for CRE carriage.

**Table 1:** Patient, clinical and healthcare facility data stratified by CRE episode type.

	Total episodes	Clinical episodes	Carriage episodes	*p*-value
	N, %	N, %	N, %	
**Number of CRE episodes**	2242 (100)	1580 (70.5)	662 (29.5)	-
**Year**				
2016	145 (6.5)	131 (8.3)	14 (2.1)	
2017	137 (6.1)	128 (8.1)	9 (1.4)	<0.001
2018	264 (11.8)	230 (14.6)	34 (5.1)	
2019	930 (41.5)	522 (33.0)	408 (61.6)	
2020	766 (34.2)	569 (36.0)	197 (29.8)	
**Sex**				
Male	1167 (52.0)	811 (51.3)	356 (53.8)	0.290
**Age category**				
Neonate (0–28 days)	218 (9.7)	48 (3.0)	170 (25.7)	
Child (>28 days– 13 years)	565 (25.2)	200 (12.6)	365 (55.1)	<0.001
Adult (>13 years)	1459 (65.1)	1332 (84.3)	127 (19.2)	
**Age at specimen collection in years, median (IQR)**	31.5 (0–52)	40 (25–57)	0 (0–2)	<0.001
**Type of healthcare encounter at specimen collection**				
Inpatient admission	2025 (90.3)	1384 (87.6)	641 (96.8)	<0.001
Outpatient visit	217 (9.7)	196 (12.4)	21 (3.2)	
**Type of healthcare facility where specimen was collected**				
Central hospital	1570 (70.0)	1069 (67.7)	501 (75.7)	
District hospital	396 (17.7)	338 (21.4)	58 (8.8)	<0.001
Regional hospital	188 (8.4)	96 (6.1)	92 (13.9)	
Specialised hospital	34 (1.5)	23 (1.5)	11 (1.7)	
Community clinic	54 (2.4)	54 (3.4)	0 (0)	
**Specimen type**				
Sterile site	523 (23.3)	523 (33.0)	0 (0)	<0.001
Non-sterile site	1719 (76.7)	1057 (66.9)	662 (100)	
**Interval from hospital admission to specimen collection in days (median, IQR)**	8 (2–19)	8 (2–20)	7 (1–17)	0.011
**Proportion of inpatients with positive CRE isolate on admission day**	336 (15.4)	225 (14.7)	111 (16.8)	0.228
**Hospital admission outcome (n = 2019)**				
Discharged	1607 (79.6)	1008 (73.1)	599 (93.6)	<0.001
Died	412 (20.4)	371 (26.9)	41 (6.4)	
**Interval between specimen collection and death in days (median, IQR)**	8 (2–21)	7 (2–19)	13 (4–30)	0.140
**CRE-attributable mortality** (death within 3 days of specimen collection), n (%)	142 (33.4)	132 (34.4)	10 (24.4)	0.198

### Species distribution

**Fig 1:**
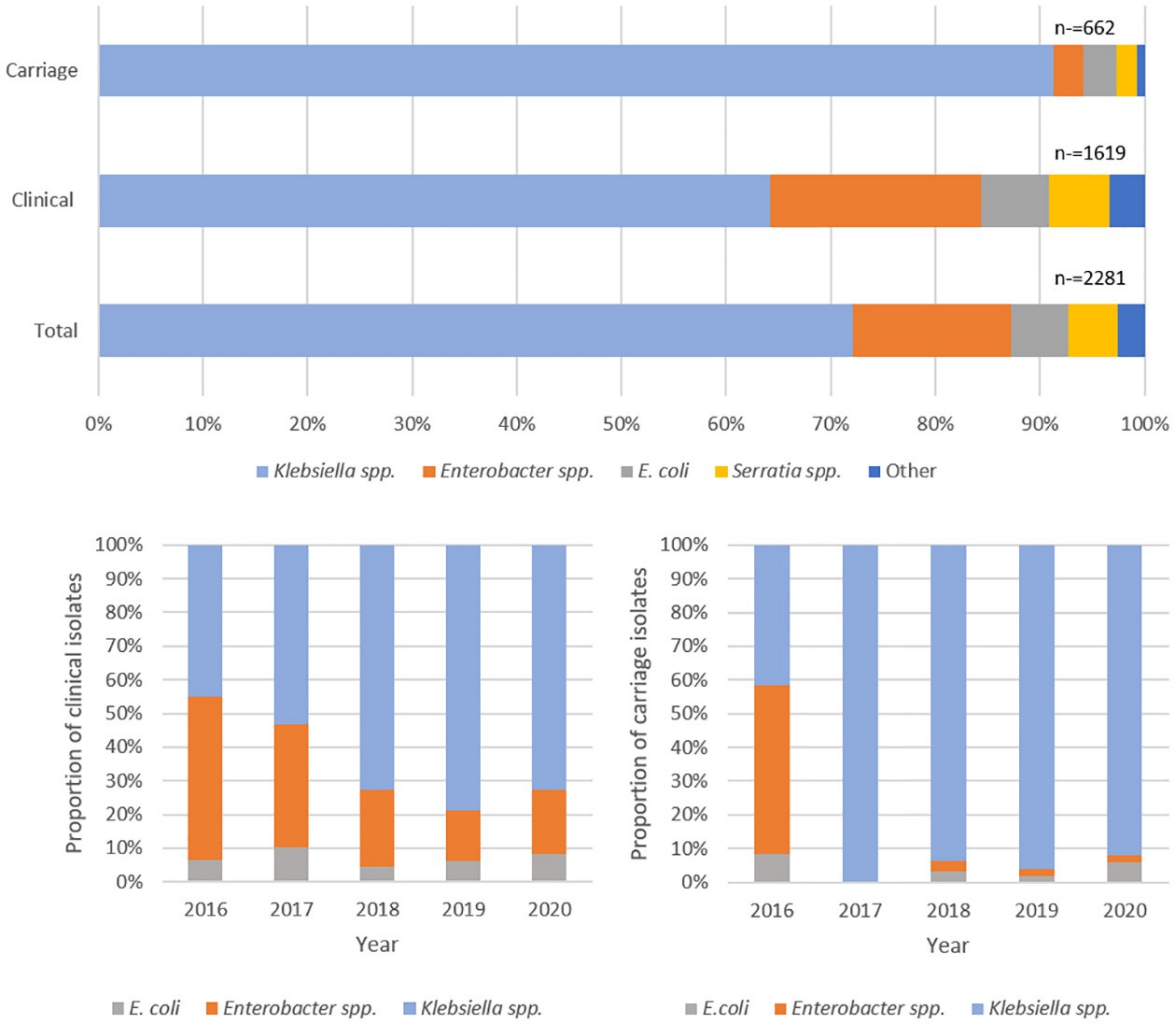
a. Spectrum of bacterial species among clinical versus carriage CRE episodes. The group “other” consists of *Citrobacter* spp., *M*. *morgannii* and *Providencia* spp. b. The distribution of the most common bacterial species between 2016 and 2020 amongst clinical and carriage isolates.

A total of 2281 isolates were reported, 71.0% from clinical episodes and 29.0% from carriage episodes. The most common CRE species isolated during the five -year period was *Klebsiella* spp. (n = 1644, 72.1%), consisting of 97.7% (n = 1606) *Klebsiella pneumoniae* and 2.3% (n = 38) *Klebsiella oxytoca*. This was followed by *Enterobacter cloacae* complex (n = 345, 15.1%), *E*. *coli* (n = 126, 5.5%) and *S*. *marcescens* (n = 107, 4.7%). The species distribution amongst clinical and carriage isolates were similar, with *Klebsiella* spp. predominating, but to a larger extent amongst clinical isolates (Fig 1a). There was an increase in the proportion of *Klebsiella* spp. in both clinical and carriage episodes and a decrease in *Enterobacter* spp. during the study period (p<0.001). There was very little change in the proportion of *E*. *coli* isolates from 2016 to 2020 (Fig 1b).

### Antibiotic susceptibility patterns of CRE isolates

**Fig 2:**
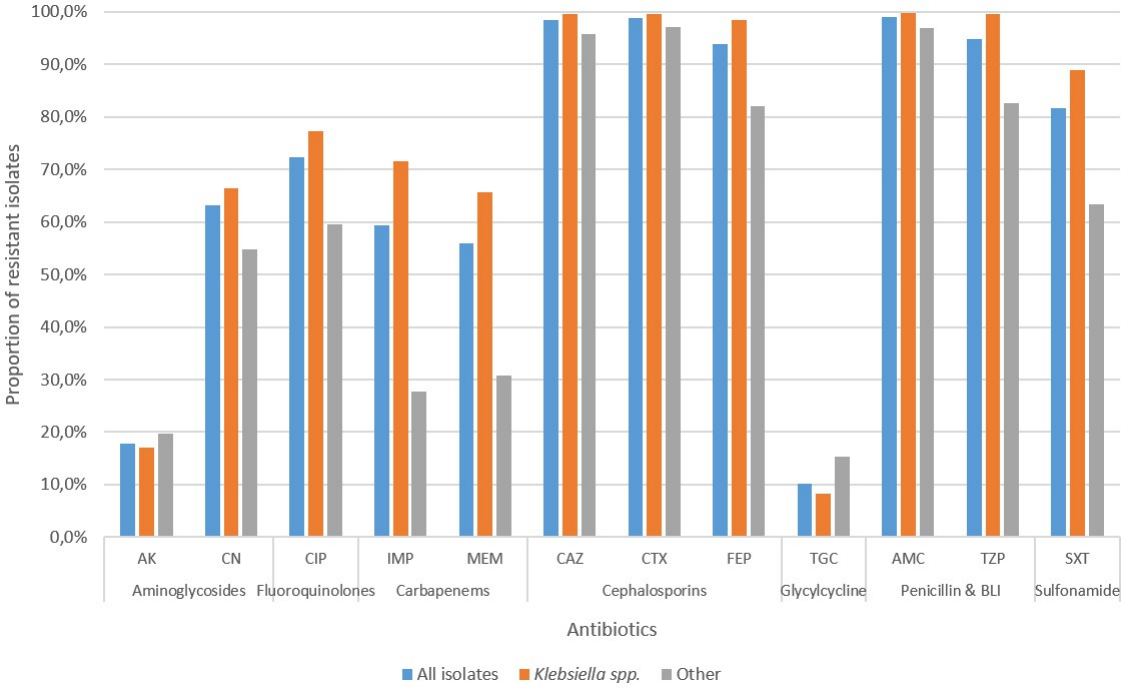
Antibiotic susceptibility patterns of all CRE isolates in comparison to *Klebsiella* spp. and non-*Klebsiella* spp (other) isolates from the Western Cape from 2016 to 2020.

The CRE isolates were least resistant to tigecycline (10.2%) and amikacin (17.8%). The proportions of isolates that were non-susceptible (resistant or intermediate) to imipenem and meropenem were 77.6% and 74.6%, respectively. Resistance to other beta-lactams was uniformly high, exceeding 98% (Fig 2) (p<0.001). Due to variability in colistin susceptibility testing methodology between laboratories as well as over time (initially tested on Vitek 2, and later referred for broth microdilution), data for colistin is not shown.

### Clinical characteristics of all episodes

Most CRE episodes occurred in hospitalised patients (n = 2025; 90.3%), with 70.5% of these episodes representing clinical cases (Table 1), of which most occurred at central hospitals (n = 1570, 70.0%, of which 67.7% were clinical episodes), followed by district hospitals (n = 396, 17.7%, of which 85.4% were clinical episodes) and regional hospitals (n = 188, 8.4%, of which 51.1% were clinical episodes).

Most clinical episodes occurred in adult patients (65.1%), whereas most carriage episodes occurred in children (55.1%) and neonates (25.7%). The predominant specimen types yielding CRE isolates submitted from patients with clinical episodes were urine (42.3%) and blood cultures (18.2%). The remaining 39.5% was made up of 10.1% each of superficial swabs and respiratory samples, fluid aspirate (6.5%), tissue, (5.8%), catheter (4.2%), abscess (2.5%) and faecal specimen (0.3%) (S1 Table). The median time from hospital admission to collection of the CRE positive specimen was 8 days (IQR, 2–19). Fifteen percent of the episodes (n = 336) were CRE positive on the day of admission, based on a positive clinical or carriage isolate, with no significant difference between clinical and carriage (*p* = 0.228) (Table 1).

Outcome data were available for 2019 CRE episodes (92.1%) amongst admitted patients, with 1379 (66.8%) being clinical CRE episodes (Outcome was only analysed for inpatient episodes with known outcome. Outcome data was not available for six episodes (five clinical, one carriage)). Overall, 412 (20.4%) deaths were recorded, with significantly higher mortality among patients with clinical episodes than those with carriage episodes (371/1379 [26.9%] vs 41/640 [6.4%]; *p* = <0.001) (Table 1).

Multivariable logistic regression showed that female sex, age (specifically adult patients), isolation of CRE from a sterile specimen and a > 3-day interval from hospital admission to specimen collection were associated with in-hospital mortality (Table 2).

**Table 2:** Factors associated with in-hospital mortality among patients with clinical CRE infection (n = 1379).

Variable	Discharged n = 1008	Died n = 371	Bivariable analysis (*p*-value)	Multivariable analysis	Adjusted odds ratio (aOR) (95% confidence interval)
	(73.1%)	(26.9%)		(*p*-value)	
**Time period (years)**					
2016–2018	314 (76.0)	99 (24.0)			Reference
2019–2020	694 (71.8)	272 (28.2)	0.108	0.223	1.19 (0.90–1.56)
**Sex**					
Male	546 (75.3)	179 (24.7)			Reference
Female	462 (70.6)	192 (29.4)	0.051	0.008	1.40 (1.09–1.80)
**Age category**					
Neonate/Child	178 (80.5)	43 (19.5)			Reference
Adult	830 (71.7)	328 (28.3)	0.006	0.004	1.76 (1.20–2.57)
**Hospital type**					Reference
Regional/District/Specialised	302 (77.8)	86 (22.2)			
Central	706 (71.2)	285 (28.8)	0.013	0.263	1.18 (0.88–1.59)
**Ward type**					
Medical	729 (75.4)	238 (24.6)			Reference
Surgical	279 (67.7)	133 (32.8)	0.003	0.331	1.14 (0.87–1.50)
**Species type**					
All other species*	344 (74.1)	121 (25.9)			Reference
*Klebsiella* species	662 (72.6)	250 (27.4)	0.552	0.600	1.07 (0.82–1.40)
**Specimen type**					
Sterile sites	306 (61.3)	193 (38.7)			Reference
Non-sterile sites	702 (79.8)	178 (20.2)	<0.001	<0.001	0.41 (0.32–0.53)
**Interval from admission to CRE specimen collection**					
<3 days	232 (80.8)	55 (19.2)			Reference
≥ 3 days	776 (71.1)	316 (28.9)	0.001	0.010	1.56 (1.11–2.18)

## Discussion

CRE infections have become a critical problem and a significant threat to health globally, including in South African healthcare facilities [12]. In the Western Cape province, between 2016 and 2020, clinical episodes increased substantially, particularly between 2018 and 2019. Other epidemiologic descriptions of CRE prevalence trends in South Africa (2019–2020) also demonstrated increasing rates in four provinces, including the Western Cape, where the proportion of reported cases had doubled [12]. A contributing factor to the peak in CRE episodes in 2019 was the declaration of a CRE outbreak at one of the central hospital neonatal units [20], which led to intensified CRE screening and surveillance practices. The higher number of CRE carriage cases among children and neonates is attributed to the heightened screening practices in those platforms/ wards, especially during and following the CRE outbreak. More screening was done in these wards as this population is usually more vulnerable due to their underdeveloped immune systems, thus making them more susceptible to infections. Screening was also done to identify potential carriage of CRE in order to separate colonised patients from non-colonised patients to prevent further spread of CRE within and between the wards. There was no clear impact of the COVID pandemic in 2020 on clinical CRE episodes, with similar numbers reported in 2019 and 2020. Data from one of the tertiary hospitals in Cape Town likewise showed no difference in incidence of Enterobacterales bacteraemia, and no difference in carbapenem resistance among Enterobacterales, comparing the 12 months before COVID to the first two waves of the pandemic in 2020. The reduction in carriage cases during 2020 may represent less intensive screening practises driven at least in part by reallocation of resources and priorities during the early stages of the COVID pandemic. Over the 5-year study period, the crude in-hospital CRE-associated mortality rate was 26.9%. Although this is worryingly high, it is lower than mortality rates of 38% previously reported in Colombia [21] and 42.2% in Korea [22] in patients with CRE infections.

*Klebsiella* spp., specifically *K*. *pneumoniae*, dominated the CRE species during this period. *K*. *pneumoniae* accounted for more than half of the CRE cases. This was followed by *Enterobacter* spp. and *E*. *coli*. Their distribution from 2016 to 2020 showed that *Klebsiella* spp., increased throughout that study period in both clinical and carriage episodes, whereas, *E*. *coli* remained somewhat steady. Interestingly, the number of *Enterobacter* spp. decreased, especially among carriage episodes. A closer look at the population showed that the majority of *Enterobacter* spp. were from adult patients, with no isolates from neonatal patients. This could be due to the CRE screening practices during the 2019 CRE outbreak where most of the screening was on the neonatal and paediatric platforms and most of the clinical cases/infections were recorded among adult patients. Globally, *K*. *pneumoniae* remains the dominant pathogen among patients with CRE infections. The 2021 surveillance of antimicrobial resistance in Europe reported that in the European Economic area (EEA) region, resistance to third-generation cephalosporins and carbapenems was generally higher in *K*. *pneumoniae* than *E*. *coli*. While carbapenem resistance was rare in *E*. *coli* in most countries, 33% of the countries reported resistance rates of 25% or higher in *K*. *pneumoniae* [23]. *K*. *pneumoniae* infections have increased during the last decade throughout the world [24] with CR *K*. *pneumoniae* remaining the dominant CRE in South Africa [8, 25]. This is concerning as this pathogen often harbours mobile genetic elements such as plasmids through which carbapenem resistance as well as resistance to other classes of antibiotics is disseminated. They may spread within and between wards and hospitals, and to other bacterial species [26].

Almost three quarters of all CRE isolates, and half of *Klebsiella spp*. were susceptible to tigecycline, with a smaller proportion of isolates susceptible to amikacin and gentamicin. Up to a quarter of CRE isolates retained susceptibility to imipenem and meropenem, despite being ertapenem resistant. This has been reported in a previous study where the loss of the outer membrane OmpK35 was shown to account for resistance to ertapenem in *K*. *pneumoniae* [27]. This may also be attributed to specific carbapenemase enzymes (such as the OXA-like enzymes) where isolates do sometimes appear phenotypically susceptible to some of the carbapenems [28]. 70.6% of all isolates were resistant to cephalosporins, in agreement with findings of a previous study that reported total resistance to all 3^rd^ and 4^th^ generation cephalosporins tested [29]. This could be due to the ability of carbapenemases to also hydrolyse cephalosporins or to concomitant presence of additional enzymes such as ESBLs or AmpC-type [30]. Although the resistance patterns of all isolates versus those of *Klebsiella spp*. were similar, a higher proportion of *Klebsiella spp*. were resistant to cefotaxime, cefuroxime, ceftazidime, cefepime, and piperacillin-tazobactam.

This study showed that female patients, adult patients, patients from whom CRE isolates were isolated from sterile sites, and those who had a CRE isolated more than three days after admission had increased odds of in-hospital mortality. This is consistent with previous studies that showed increased mortality associated with age (patients older than 60 years) [12]. Other reported factors that have been shown to increase the odds of in-hospital mortality are comorbidities, altered mental state and previous antimicrobial use [12], however this data was not available for this study.

There was only a small proportion of patients with a positive CRE sample on admission. This and the higher mortality in patients where CRE isolates were obtained >3 days suggests that the majority of the CRE cases may be hospital acquired. This shows that patients may benefit from strengthened prevention measures, such as hand hygiene, isolation precautions, antibiotic stewardship as well as surveillance and active screening to reduce infections and consequently CRE-related mortality [31]. It is important to identify these factors to improve patient outcomes, to guide treatment strategies, enhance infection prevention and control (IPC) measures and to address the broader public health and economic implications of CRE infections. This ultimately impacts the greater fight against AMR and the improvement of health care.

This study has several limitations including the lack of access to complete patient clinical records and the retrospective nature of the study. Other limitations include the difficulties in the inclusion and exclusion of CRE cases due to duplicates (i.e one patient with multiple CRE cases). Several factors needed to be considered in the decision to include or exclude a CRE episode such as collection date, type of sample and bacterial species. Although a number of studies have described the circulating carbapenemases in the Western Cape, with the dominant ones being NDM and OXA-48-like, we did not have the ability to link specific carbapenemases to patients in our study. We were thus unable to assess the effect of carbapenemase type on outcome. Study strengths include the large sample size allowing more precise estimates and the use of robust definitions of CRE.

## Conclusions

The escalation of CRE cases in the Western Cape emphasises the growing problem of carbapenem resistance, increasing the complexity, cost of treatment and mortality in patients with CRE infection. We determined that female sex, adult patients, isolation of CRE isolate from sterile specimen and a >3 day interval from hospital admission to specimen collection is associated with mortality in patients with CRE infections. Ongoing surveillance to monitor CRE distribution, infection types and risk factors for mortality in Western Cape patients is crucial to inform both clinical care and infection prevention efforts in the province.

## Supporting information

S1 Table Sheet 1: Distribution of clinical and carriage samples among CRE episodes, Sheet 2: Western Cape healthcare facilities. (XLSX)
